# Impact of Strain and Morphology on Magnetic Properties of Fe_3_O_4_/NiO Bilayers Grown on Nb:SrTiO_3_(001) and MgO(001)

**DOI:** 10.3390/ma11071122

**Published:** 2018-06-30

**Authors:** Olga Kuschel, Nico Pathé, Tobias Schemme, Kevin Ruwisch, Jari Rodewald, Ralph Buss, Florian Bertram, Timo Kuschel, Karsten Kuepper, Joachim Wollschläger

**Affiliations:** 1 Department of Physics and Center of Physics and Chemistry of New Materials, Osnabrück University, 49076 Osnabrück, Germany; oschuckm@uos.de (O.K.); npathe@uos.de (N.P.); toschemm@uos.de (T.S.); kruwisch@uos.de (K.R.); jarodewa@uos.de (J.R.); rbuss@uos.de (R.B.); kkuepper@uos.de (K.K.); 2 Deutsches Elektronen-Synchrotron (DESY), Photon Science, 22607 Hamburg, Germany; florian.bertram@desy.de; 3 Center for Spinelectronic Materials and Devices, Department of Physics, Bielefeld University, 33615 Bielefeld, Germany; tkuschel@physik.uni-bielefeld.de

**Keywords:** magnetite, nickel oxide, strain relaxation, magnetic anisotropy

## Abstract

We present a comparative study of the morphology and structural as well as magnetic properties of crystalline Fe_3_O_4_/NiO bilayers grown on both MgO(001) and SrTiO_3_(001) substrates by reactive molecular beam epitaxy. These structures were investigated by means of X-ray photoelectron spectroscopy, low-energy electron diffraction, X-ray reflectivity and diffraction, as well as vibrating sample magnetometry. While the lattice mismatch of NiO grown on MgO(001) was only 0.8%, it was exposed to a lateral lattice mismatch of −6.9% if grown on SrTiO_3_. In the case of Fe_3_O_4_, the misfit strain on MgO(001) and SrTiO_3_(001) amounted to 0.3% and −7.5%, respectively. To clarify the relaxation process of the bilayer system, the film thicknesses of the magnetite and nickel oxide films were varied between 5 and 20 nm. While NiO films were well ordered on both substrates, Fe_3_O_4_ films grown on NiO/SrTiO_3_ exhibited a higher surface roughness as well as lower structural ordering compared to films grown on NiO/MgO. Further, NiO films grew pseudomorphic in the investigated thickness range on MgO substrates without any indication of relaxation, whereas on SrTiO_3_ the NiO films showed strong strain relaxation. Fe_3_O_4_ films also exhibited strong relaxation, even for films of 5 nm thickness on both NiO/MgO and NiO/SrTiO_3_. The magnetite layers on both substrates showed a fourfold magnetic in-plane anisotropy with magnetic easy axes pointing in $\langle 100\rangle$ directions. The coercive field was strongly enhanced for magnetite grown on NiO/SrTiO_3_ due to the higher density of structural defects, compared to magnetite grown on NiO/MgO.

## 1. Introduction

Transition metal oxides are one of the most interesting material classes, providing a huge variety of structural, magnetic, and electronic properties ranging from metallic to insulating, from ferro- to antiferromagnetic, as well as ferroelectric states [1]. Especially, thin magnetite films (Fe_3_O_4_) have attracted intensive research interest in the last decade in the field of spintronics [2] and spin caloritronics [3,4]. Due to their anticipated half-metallic behavior with complete spin polarization at the Fermi level [5] and high (bulk) Curie temperature of 858 K [6], thin magnetite films are promising candidates for room temperature spintronic devices such as highly spin-polarized electrodes for magnetic tunneling junctions [7,8,9] or spin injectors [10]. Furthermore, multilayers of magnetite and platinum show huge thermoelectric effects [11] based on the recently observed spin Seebeck effect in magnetite [12] pushing the development of more efficient thermoelectric nanodevices [13].

Magnetite crystallizes in the inverse spinel structure with a lattice constant of 8.3963 Å [6] at 300 K. At ∼120 K it undergoes a metal–insulator transition (Verwey transition) [14] accompanied by a change from cubic to monoclinic crystal symmetry [15]. The reduction of the crystal symmetry leads to a spontaneous ferroelectric polarization, and thus to multiferroicity [16,17].

In order to control the relative magnetization alignment in magnetic tunnel junctions, exchange bias effects induced by additional antiferromagnetic layers are commonly used [18]. In the case of Fe_3_O_4_ tunnel junctions, the antiferromagnetic NiO is a good candidate due to its small lattice mismatch of only 0.5% and a high Néel temperature of 523 K [19].

Nickel oxide is an insulating material with a high thermal stability. It crystallizes in a rock salt structure with a lattice constant of 4.1769 Å [20] at 300 K. It was recently shown that NiO can act as a spin current amplifier in spin Seebeck experiments, and can additionally be a spin current generator when a thermal gradient is applied [21,22,23,24], making NiO a key material for thermoelectric devices. Further, the latest studies report on a temperature-dependent sign change in the spin hall magnetoresistance for nickel oxide on ferromagnetic insulator [25,26]. Thus, there is a possibility to use it as a spin filter.

Previous works [27,28,29,30,31,32,33] have focused on the characterization of magnetite and nickel oxide films grown on MgO substrates because of the small lattice mismatch of 0.3% and 0.8%, respectively. However, it has been demonstrated that the electronic and magnetic properties of magnetite films can be modified using SrTiO_3_ substrates [34,35,36], despite the large lattice mismatch of −7.5%. One advantage of using SrTiO_3_ substrates is the possibility of doping and, thus, a tunable conductivity providing either an insulating or metallic substrate which can be used as a bottom electrode in capacitor-like structures [17]. Furthermore, Fe_3_O_4_/NiO bilayers grown on SrTiO_3_ can be used to synthesize Ni*_x_*Fe_3−*x*_O_4_ thin films by thermally induced interdiffusion with tunable magnetic and electric properties [37].

To date, most studies concerning NiO films on SrTiO_3_ have been limited to a coarse analysis of the growth [38,39], while a thorough structural characterisation is seldom reported [40]. In the case of Fe_3_O_4_/NiO bilayers on both substrates, there are a number of works on electronic structure, interfacial coupling, and magnetic characterization [41,42,43,44], whereas to the best of our knowledge there are no detailed structural studies for these bilayers on SrTiO_3_. However, the magnetic and transport characteristics of such films are sensitive to structural variations, number of defects, or stoichiometric deviations, and can be affected by the strain between film and substrate [36]. Therefore, in this work, a comprehensive structural characterization of Fe_3_O_4_/NiO bilayers of different thicknesses grown on Nb-doped SrTiO_3_(001) and for comparison on MgO(001) is presented. Additionally, these results are correlated with magnetic properties (e.g., magnetocrystalline anisotropy).

Directly after deposition, the stoichiometry in the near-surface region and the surface structure of each layer was determined in situ using X-ray photoelectron spectroscopy (XPS) and low-energy electron diffraction (LEED), respectively. The bulk structure was investigated ex situ by X-ray reflectivity (XRR) and synchrotron radiation X-ray diffraction (SR-XRD) measurements and analyzed within kinematic diffraction theory. Further, angle-dependent hysteresis loops were measured via vibrating sample magnetometry (VSM).

## 2. Materials and Methods

Preparation and in situ characterization of the thin oxide films were carried out in an interconnected ultrahigh vacuum (UHV) system at a base pressure of 10^−8^ mbar in the deposition chamber and 10^−10^ mbar in the analysis chamber. Epitaxial Fe_3_O_4_/NiO ultra-thin bilayer systems with thicknesses between 5 nm and 20 nm were grown via reactive molecular beam epitaxy (RMBE) on 0.05% Nb-doped SrTiO_3_(001) or on MgO(001) single crystalline substrates. Prior to deposition, the substrates were annealed at 400 °C in 1 × 10^−4^ mbar O_2_ atmosphere for 1 h in order to remove carbon contamination and get well-defined surfaces. Subsequently, nickel oxide and magnetite films were deposited by thermal evaporation from pure metal rods in 1 × 10^−5^ mbar and 5 × 10^−6^ mbar oxygen atmosphere, respectively. Deposition was performed at 250 °C substrate temperature using deposition rates of 0.01 nm/s for nickel oxide films and 0.25 nm/s for magnetite films, as controlled by a quartz microbalance adjacent to the evaporation source. The resulting film thicknesses were determined later on ex situ by XRR (Panalytical, Philips X’Pert Pro, Almelo, The Netherlands) Crystal surface quality and near-surface stoichiometry were controlled in situ after each preparation step by LEED (ErLEED 150, SPECS, Berlin, Germany) and XPS (SPECS, Berlin, Germany) using an Al K*α* (h*ν* = 1486.6 eV) radiation source and a Phoibos HSA 150 hemispherical analyzer.

After transport under ambient conditions, XRR and XRD experiments were carried out ex situ for structural characterization of the films. XRR measurements were performed in θ–2θ geometry using a lab based diffractometer equipped with a Cu K${}_{\alpha }$ anode. An in-house developed fitting tool based on the Parratt algorithm [45] using Névot-Croce [46] roughness profiles was applied for the analysis of the XRR curves. For XRD synchrotron based radiation sources at the MaXLab beamline I811 (MaXLab, Lund, Sweden) and at the Swiss Light Source beamline X04SA (Paul Scherrer Institute, Villigen, Switzerland) were used. Both beamlines are equipped with (2S + 3D) type diffractometers and Pilatus pixel area detectors for data collection. The XRD data were recorded in θ–2θ geometry at an energy of 12.4 keV and analyzed within the kinematic diffraction theory [47] that is implemented in our in-house developed fitting tool.

In addition, magnetization curves were measured at room temperature for several in-plane directions of the samples by varying the magnetic field *μ*_0_*H* between −300 mT and +300 mT, using a VSM (Lakeshore, Model 7407, Westerville, OH, USA). The magnetization loops were corrected by subtracting the diamagnetic contribution from the substrates.

## 3. Results

### 3.1. LEED/XPS

**Figure 1:**
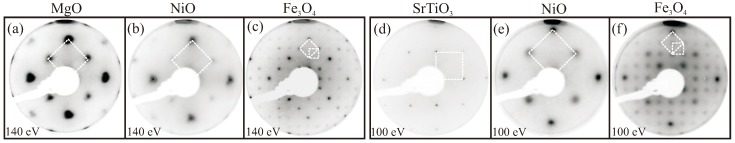
Low-energy electron diffraction (LEED) pattern recorded at 140 eV for (**a**) pure MgO(001) surface; (**b**) 11.9 nm NiO film on MgO(001); and (**c**) 21.5 nm Fe_3_O_4_ on NiO/MgO(001). The LEED pattern taken at 100 eV of a pure SrTiO_3_ surface, a 10.4 nm NiO film on SrTiO_3_(001), and 20.7 nm Fe_3_O_4_ on NiO/SrTiO_3_(001) are depicted in (**d**–**f**), respectively. The larger white squares indicate the (1 × 1) structure of the reciprocal unit cell of the respective surfaces, while the smaller white squares in (c) and (f) indicate the $(\sqrt{2}\times \sqrt{2})R45^{\circ }$ superstructure unit cell of magnetite.

Figure 1a,d presents the LEED patterns of the cleaned MgO(001) and SrTiO_3_(001) surfaces, respectively. All as-prepared NiO and Fe_3_O_4_ films showed similar LEED patterns on the respective substrate for all investigated thicknesses ranging from 5 nm to 20 nm. Thus, only patterns of a ∼20 nm Fe_3_O_4_ and a ∼10 nm NiO film on MgO and SrTiO_3_ are shown as examples in Figure 1. The intensity variations in all recorded patterns were due to dynamical scattering for electron diffraction, and will not be considered further. Instead, we focus on the symmetry of the diffracted pattern and the sharpness of the diffraction spots.

Clear (1 × 1) structures corresponding to the square unit cells of MgO(001) and SrTiO_3_(001) surfaces could be seen (cf. Figure 1a,d). Due to the rock salt structure of MgO, the reciprocal unit vectors of the MgO(001) surface point in [110] and [$\bar{1}10$] directions, forming a quadratic reciprocal unit cell. The reciprocal unit vectors of the (001) surface of the perovskite SrTiO_3_ point in [100] and [010] directions, also forming a quadratic unit cell. Consequently, the reciprocal surface unit vectors of MgO(001) are ∼$\sqrt{2}$ times larger than those of SrTiO_3_(001).

In diffraction patterns, a random arrangement of point defects leads to an increased background, while line defects (e.g., domain boundaries) result in a broadening of the diffraction spots [48]. To obtain not only qualitative but also quantitative information on the defect density, the full width of half maximum (FWHM) of the diffraction spots was determined at 140 eV, taking into account the instrumental broadening of the LEED instrument.

The SrTiO_3_ pattern exhibited sharp and intense diffraction spots. Analysis of the FWHM of the (11) diffraction peaks yielded a line defect density of (0.11 ± 0.02) nm^−2^. In contrast, the spots of the MgO substrate were broadened due to charging effects. Thus, it was not possible to determine a value for the defect density of the substrate here. The diffuse background was quite low in both patterns, pointing to clean surfaces and negligible point defects. Additionally, XPS measurements of both substrates showed no carbon contamination, indicating chemically clean surfaces.

After the deposition of NiO, the LEED patterns also exhibited a (1 × 1) structure related to the square symmetry of the NiO(001) surface for both substrates (cf. Figure 1b,e). As mentioned above, due to the rock salt structure, the reciprocal unit vectors of the NiO(001) surface point in [110] and [$\bar{1}10$] directions and are consequently ∼$\sqrt{2}$ times larger than the surface unit cell of SrTiO_3_ in reciprocal space. Due to the very similar lattice constants of NiO(001) and MgO(001), the diffraction spots were located at almost identical positions. A broadening of the diffraction spots compared to the pattern of the SrTiO_3_ substrate was clearly visible, indicating an increase of the defect density. Analyzing the FWHM of the (10) surface diffraction spots, we obtained densities of line defects of (0.8 ± 0.1) nm^−2^ and (1.1 ± 0.2) nm^−2^ for the NiO/MgO and NiO/SrTiO_3_, respectively. The slightly larger broadening of the diffraction spots for NiO/SrTiO_3_ compared to the diffraction spots of the NiO/MgO surface can be related to the formation of more structural defects (e.g., domain boundaries), induced by the higher lattice misfit of NiO(001) on SrTiO_3_(001). Additionally, both patterns showed a negligible background intensity of the NiO(001) surface, pointing to a small amount of point defects.

The LEED images of Fe_3_O_4_ obtained after deposition on NiO/MgO(001) and NiO/SrTiO_3_(001) showed similar diffraction patterns with a square symmetry (cf. Figure 1c,f). Clear diffraction spots with half-peak distance compared to the NiO(001) surface indicated an approximately doubled lattice constant in real space due to the almost-doubled cubic lattice constant of Fe_3_O_4_ compared to the other oxides used here. Furthermore, an additional ($(\sqrt{2}\times \sqrt{2})R45^{\circ }$ superstructure appeared, which is characteristic for a well-ordered magnetite surface [49,50,51,52]. This superstructure is not observed for maghemite (Fe_2_O_3_), which has a very similar surface lattice constant. Therefore, we assume the formation of well-ordered stoichiometric magnetite films. However, the diffraction spots of the magnetite film grown on NiO/MgO were sharper than for the growth on NiO/SrTiO_3_, indicating a better ordering and less domain boundaries. For the density of line defects of the Fe_3_O_4_ films, values of (1.3 ± 0.2) nm^−2^ and (0.14 ± 0.02) nm^−2^ were obtained for the growth on NiO/SrTiO_3_(001) and NiO/MgO(001), respectively, analyzing the FWHM of the (20) surface diffraction spots.

In summary, the LEED patterns of the Fe_3_O_4_/NiO bilayer systems confirmed a crystalline cube-on-cube growth of both NiO and Fe_3_O_4_ films on MgO(001), as well as on SrTiO_3_(001). The films grown on MgO substrates exhibited a higher crystalline quality and less surface defects compared to the bilayers grown on SrTiO_3_.

**Figure 2:**
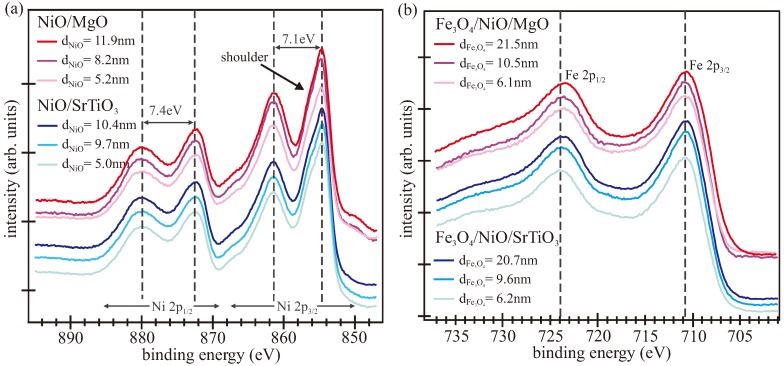
X-ray photoelectron spectra of (**a**) Ni 2p region for the as-prepared NiO films on MgO(001) and SrTiO_3_; (**b**) Fe 2p region for the as-prepared Fe_3_O_4_ films on NiO/MgO(001) and NiO/SrTiO_3_.

XPS measurements were made directly after deposition of the films to determine the stoichiometry and the valence state of the cation species. Figure 2a shows the XP spectra of the Ni 2p region after the deposition of nickel oxide and before the deposition of iron oxide. All spectra of the Ni 2p core level revealed Ni 2p_3/2_ and Ni 2p_1/2_ peaks at binding energies of 854.6 eV and 872.5 eV, respectively, and two intense satellite structures at about 7 eV higher binding energies. Since these values agree well with the binding energies reported in the literature for a Ni^2+^ valence state in NiO stoichiometry [53,54], we assume that the oxide films were stoichiometric and had negligible point defects (e.g., oxygen vacancies). Additionally, there was a shoulder ∼1.5 eV above the Ni 2p_3/2_ peak, which has been reported to be typical for NiO [55,56]. Thus, the shape of all spectra was comparable to that of NiO bulk crystal [54,57,58].

The Fe 2p photoelectron spectra of the iron oxide films as prepared on top of the NiO films are presented in Figure 2b. From the position and shape of the Fe 2p peaks, one can obtain information about the iron oxidation state and the stoichiometry. All recorded spectra exhibited the same shape, with main peaks located at binding energies of 710.6 eV and 723.6 eV for Fe 2p_3/2_ and Fe 2p_1/2_, respectively. These binding energies of the core levels correspond to well-known values of Fe_3_O_4_ from the literature [59]. Additionally, in contrast to wüstite (FeO) and maghemite (Fe_2_O_3_), no apparent charge transfer satellites can be observed between the two main peaks due to their overlap [59,60]. Consequently, the shape and binding energies of the Fe 2p spectra confirmed a mixed Fe^2+^/Fe^3+^ valence and pointed to a Fe_3_O_4_ stoichiometry for all prepared iron oxide films. Thus, both XPS and LEED measurements demonstrated that the bilayer structures on both kind of substrates consisted of crystalline stoichiometric NiO and Fe_3_O_4_ films.

### 3.2. XRR/XRD

**Figure 3:**
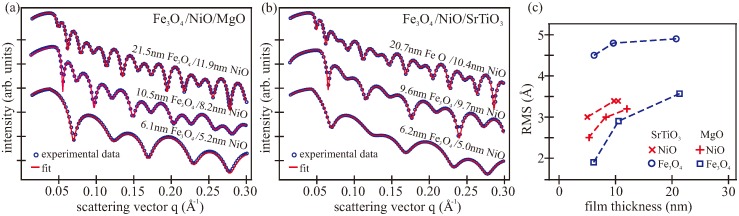
X-ray reflectivity (XRR) measurements and the calculated intensities of the bilayers on (**a**) MgO and (**b**) SrTiO_3_ substrates; (**c**) Fe_3_O_4_ surface and Fe_3_O_4_/NiO interface roughnesses obtained from the XRR measurements.

XRR and XRD experiments were performed ex situ to determine the structural parameters of the bilayers (e.g., film thicknesses and vertical lattice distances). Figure 3a,b shows the measured reflectivity curves and the corresponding calculated reflectivity curves after optimizing the structural parameters. In addition, the obtained thicknesses of all studied bilayers are presented. Clear intensity oscillations with beating effects were visible for all samples, indicating double-layer structures and flat homogenous films with small interface and surface roughness.

The applied calculation model consists of a layer of iron oxide on top of a nickel oxide layer on MgO or SrTiO_3_ substrate. All fitted curves agreed excellently with the experimental data using literature values for the dispersion $\delta_{{\mathrm{Fe}}_{3}O_{4}}$ = 1.53 × 10^−5^ and $\delta_{\mathrm{NiO}}$ = 1.89 × 10^−5^ [61]. This indicates a small defect density (e.g., oxygen vacancies), which is in accordance with the XPS results.

Additionally, the roughnesses of the films were determined and are presented in Figure 3c. Here, all films featured an increase of the surface and interface roughness with increasing film thickness. This effect can be attributed to kinetic roughening of the films during growth and to the progressing relaxation process [62]. The nickel oxide films exhibited similar roughnesses of $\sigma_{\mathrm{NiO}}$ = 2.5–3.5 Å on both substrates, with a small increase for thicker films. The roughness of the Fe_3_O_4_ on NiO/MgO increased more drastically, while the magnetite films deposited on NiO/SrTiO_3_ showed nearly constant roughness with initially almost doubled values compared to the magnetite films on NiO/MgO. This behavior is likely caused by high lattice misfit and the resulting relaxation process. This is in accordance with the broadened diffraction spots of the Fe_3_O_4_ films on NiO/SrTiO_3_ observed in the LEED pattern (cf. Figure 1).

**Figure 4:**
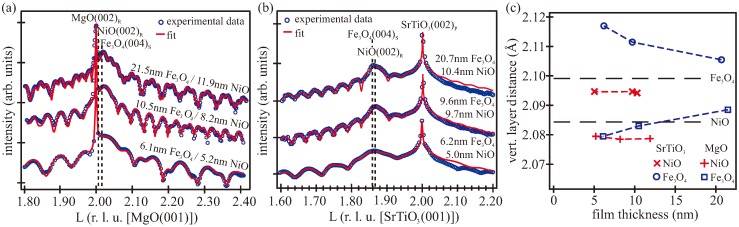
X-ray diffraction (XRD) measurement along the (00*L*) crystal truncation rod (CTR) (**a**) of the Fe_3_O_4_/NiO/MgO samples and (**b**) of the Fe_3_O_4_/NiO bilayers on SrTiO_3_. The calculated intensity distribution using the kinematic approximation is shown in red. (**c**) Vertical layer distance of nickel oxide and magnetite grown on MgO(001) and SrTiO_3_(001), dependent on the film thickness. The dashed lines denote the fully relaxed bulk values of magnetite and nickel oxide.

Figure 4a,b presents the SR-XRD measurements of the (00*L*) crystal truncation rod (CTR) compared to intensities calculated by kinematic diffraction theory of the Fe_3_O_4_/NiO bilayers on MgO(001) and SrTiO_3_(001), respectively. Here, the bulk nomenclature of the reciprocal space was used, where *L* = $c\;K_{⊥}$/(2π) in reciprocal lattice units (r.l.u.) denotes the vertical scattering vector $K_{⊥}$ scaled to the Bragg condition 2 π/*c* ($c_{\mathrm{MgO}}$ = 4.2117 Å, $c_{{\mathrm{SrTiO}}_{3}}$ = 3.905 Å). The diffraction data revealed an epitaxial (001)-oriented growth of NiO and Fe_3_O_4_ on both substrates. Due to the almost-doubled lattice constant of magnetite compared to both MgO and NiO and the resulting lateral tensile strain, the (004)*_S_* spinel reflection was located at higher *L* values compared to MgO and close to the (002)*_R_* bulk reflection of a rock salt structure. On SrTiO_3_, both nickel oxide and magnetite exhibited a large lattice misfit and were laterally compressively strained. Thus, the (004)*_S_* reflection of magnetite and (002)*_R_* reflection of NiO were at lower *L* values compared to SrTiO_3_ and were well separated from the (002)*_P_* perovskite reflection of SrTiO_3_. Here, the indexes *R*, *S*, and *P* indicate bulk indexing for rock salt, spinel, and perovskite types, respectively.

For all bilayers grown on MgO, the measurements showed a sharp peak at *L* = 2 originating from the diffraction at the MgO substrate lattice (cf. Figure 4a). Additionally, broad and rather intense features located at *L*∼2.02 accompanied by strong Laue oscillations were visible due to the finite thickness of the iron and nickel oxide films. The well-pronounced intensity oscillations with two superposed partial oscillations clearly showed a periodicity of two layers of different thickness, indicating a high crystalline ordering and homogenous thicknesses of both films—magnetite and nickel oxide. This is in accordance with the results seen in the XRR measurements.

In the case of bilayers grown on SrTiO_3_, the (00*L*) rod also showed a sharp substrate peak at *L* = 2 and Laue oscillations due to crystalline magnetite and nickel oxide films (cf. Figure 4b). Here, the Bragg peaks originating from the iron and nickel oxide were located at *L* ∼ 1.86 and were broadened due to the finite film thicknesses. Upon closer inspection, the Laue oscillations also showed a periodicity of two layers, whereby the damping of the oscillation originating from the magnetite surface increased with increasing magnetite thickness due to increasing roughness (cf. Figure 3c). This result agrees well with LEED and XRR results shown above.

Due to the small lattice mismatch between Fe_3_O_4_ and NiO, a separation of the Bragg peaks originating from the respective film is not visible by eye. Complete data analysis using kinematic diffraction theory was performed to obtain the vertical layer distance of the respective oxide film. Within the calculation, the atomic form factors of oxygen, nickel, and iron atoms arranged in a bulk structure were kept constant while the vertical size of the unit cell was varied. Interface roughness was modeled with a Gaussian variation of the height as implemented for XRR by the Névot–Croce model [46]. The applied models consist of a homogenous Fe_3_O_4_/NiO bilayer on top of the respective substrate. This structural model involving the number of layers coincides with the layer model and the film thicknesses obtained from XRR calculations. The obtained vertical layer distances ($c_{\mathrm{NiO}}$/2 for NiO and $c_{{\mathrm{Fe}}_{3}O_{4}}$/4 for Fe_3_O_4_) are shown in Figure 4c.

The dashed lines mark the bulk values of the magnetite and nickel oxide. Due to the larger unit cell of MgO(001), pseudomorphic growth of NiO on MgO resulted in an expansion of the NiO unit cell in the lateral direction, and thus a vertical compression, and consequently a smaller vertical lattice distance. Exactly the opposite was expected in the case of NiO grown on SrTiO_3_(001), due to the smaller bulk unit cell of SrTiO_3_ compared to NiO. Thus, the vertical lattice distance of NiO was larger than the bulk value, as observed in the experiment.

For the NiO layers on MgO, the vertical layer distance exhibited a compressive strain (2.078 Å) due to lateral tension, and showed no dependence on the NiO thickness in the investigated range (cf. Figure 4c). In the case of bilayers grown on SrTiO_3_, the vertical lattice distance of NiO (2.095 Å) pointed to tensile strain as a result of the lateral compression. Further, there was no dependence on the NiO thickness.

However, the situation was different for the relaxation of the magnetite films. Due to pseudomorphic growth of NiO on MgO, the vertical layer distance of Fe_3_O_4_ grown on top of NiO/MgO was also slightly compressively strained but relaxed to higher values with increasing magnetite thickness. Its value relaxed from 2.0795 Å for the 6.1 nm thick magnetite film to 2.0885 Å for the thickest magnetite film. A strong relaxation with increasing film thickness of the magnetite could also be seen for magnetite films grown on NiO/SrTiO_3_. The vertical lattice distance of Fe_3_O_4_ on NiO/SrTiO_3_ was exposed to heavy tensile strain and decreased rapidly from 2.117 Å for the thinnest film to 2.106 Å for the 20.7 nm thick magnetite film.

### 3.3. VSM

**Figure 5:**
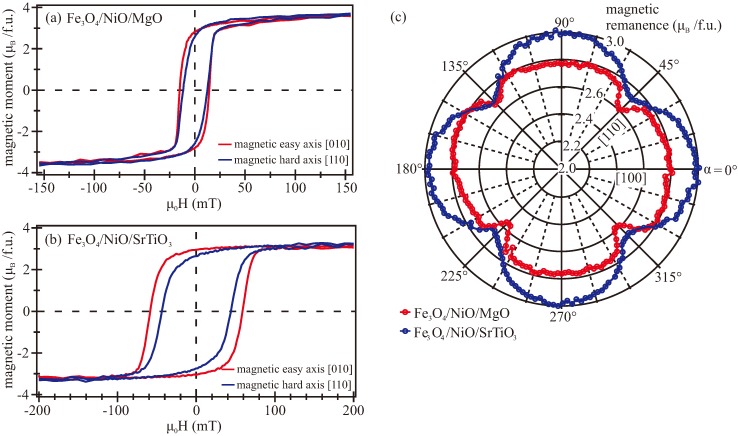
Vibrating sample magnetometry (VSM) magnetization curves of magnetic easy and hard directions for (**a**) 21.5 nm-thick Fe_3_O_4_ film on NiO/MgO and (**b**) 20.7 nm-thick Fe_3_O_4_ film on NiO/SrTiO_3_. (**c**) Polar plot of the magnetic remanence depending on the azimuthal sample angle α of a 21.5 nm-thick Fe_3_O_4_ film on NiO/MgO (red) and 20.7 nm-thick Fe_3_O_4_ film on NiO/SrTiO_3_ (blue).

As an example, the magnetic properties of the two thickest magnetite films on NiO/MgO and NiO/SrTiO_3_ were studied by means of VSM. The magnetization curves were measured for different azimuthal sample directions α between the substrate [100] direction and the applied magnetic field. Figure 5a,b shows the magnetic moment per f.u.(formula unit) as a function of the magnetic field for the bilayers on MgO and SrTiO_3_, respectively, for two different directions of the external magnetic field. For both samples, a typical ferro(i)magnetic behavior was observed. Here, the red curves recorded with the magnetic field applied in the [010] direction of the substrates represent magnetic easy axes with a high magnetic remanence and coercive fields. The blue curves recorded with the magnetic field applied in the [110] direction exhibit the magnetic behavior of a magnetic hard axis due to a lower strength of the coercive field and a smaller magnetic remanence. However, from magnetic saturation to magnetic remanence, neither investigated sample was in a monodomain state. This can also be concluded from the squareness (magnetic remanence value devided by the saturation magnetization) for the field loop of the magnetic easy direction which is below one. This effect is probably originated in the presence of antiphase boundaries that pin the magnetic moments in different directions and, thus, support multidomain states rather than monodomain states, even for the case of having the magnetization aligned in the magnetic easy direction.

The Fe_3_O_4_ film on NiO/SrTiO_3_ showed an enhanced coercive field compared to the magnetite film grown on NiO/MgO. One possible reason could be a higher density of grain boundaries due to the relaxation process, which supports pinned multidomain states that need larger magnetic fields to be switched. This is consistent with the weaker structural quality (e.g., high roughness, broad diffraction peaks) seen in the LEED, XRR, and XRD measurements. Further, the saturation magnetization of the Fe_3_O_4_ film grown on NiO/MgO amounted to (3.7 ± 0.3) $\mu_{B}$/f.u., and was rather close to the literature value of 4.07 $\mu_{B}$/f.u. [5,63]. In contrast, magnetite on NiO/SrTiO_3_ showed a lower magnetic moment of (3.3 ± 0.3) $\mu_{B}$/f.u., which may result from the antiferromagnetic coupling in the vicinity of anti-phase domain boundaries (APBs) [64].

The remanent magnetization as a function of azimuthal sample angle α is shown in Figure 5c for both investigated samples. The maxima of the magnetic remanence pointed in $\langle 100\rangle$ directions for both Fe_3_O_4_ films on NiO/MgO and NiO/SrTiO_3_, indicating the magnetic easy directions. Consequently, the magnetic hard axes were located in $\langle 110\rangle$ directions.

## 4. Discussion

XPS measurements taken directly after deposition revealed stoichiometric Fe_3_O_4_ and NiO on both substrates, independent of the film thicknesses. Due to the limited mean free path of the electrons, only the near-surface region (∼5 nm) of the layers could be characterized. No evidence for the formation of non-stoichiometric magnetite was observed in this region. Pilard et al. found a 1.5 nm-thick NiFe_2_O_4_ interfacial layer after depositing NiO above 610 °C on Fe_3_O_4_ [41]. Within the XPS measurements presented here, the interfacial region could be detected only for the thinnest magnetite films, showing spectral shape and binding energies typical for Ni^2+^ in NiO stoichiometry. Thus, there was no evidence for the formation of NiFe_2_O_4_, due to the lower growth temperature.

Hard X-ray photoelectron spectroscopy (HAXPES) and X-ray magnetic circular dichroism (XMCD) measurements [42] of the same samples recorded after transport under ambient conditions showed small traces of Fe^3+^ excess on the surface of the bilayers grown on SrTiO_3_. However, in deeper layers and at the interface, the presence of stoichiometric NiO and Fe_3_O_4_ was confirmed, excluding the formation of NiFe_2_O_4_ clusters or any interfacial layer also for thicker Fe_3_O_4_ films [42]. Consequently, very thin magnetite films tend to form maghemite at the surface after exposure to ambient air whereas thicker films seem to be more stable, as reported previously by Fleischer el al. [65]. Since in situ XPS and LEED measurements taken after preparation under UHV conditions showed no evidence for maghemite, a capping layer deposited directly after growth could prevent the possible oxidation process in the upper layers.

In situ LEED measurements also verified the Fe_3_O_4_ stoichiometry of the iron oxide film showing the typical $(\sqrt{2}\times \sqrt{2})R45^{\circ }$ superstructure of the magnetite surface for all investigated films. Further, NiO films on both substrates exhibited the expected (1×1) pattern due to the rock salt crystal structure. The diffraction spots of the magnetite and NiO films grown on SrTiO_3_ were slightly broadened compared to the films grown on MgO, indicating the formation of more surface defects due to the high lattice misfit. Surface roughnesses obtained from the XRR analysis exhibited higher values for all films grown on SrTiO_3_. While the roughness of the nickel oxide films deposited on SrTiO_3_ was only about 0.5 Å higher than after deposition on MgO, the magnetite films on NiO/SrTiO_3_ initially showed almost doubled values compared to the magnetite films on NiO/MgO. This result is consistent with the higher value for the defect density of Fe_3_O_4_/NiO/SrTiO_3_ obtained from the LEED pattern analysis. Nevertheless, the XRR measurements provided distinct intensity oscillations, indicating double layer structures and homogenous film thicknesses. Thus, the two layers did not intermix during the deposition process.

The entire structure of the samples was investigated by XRD measurements of the specular CTR. For all samples, the thickness determined by XRR agreed well with the number of layers obtained from XRD analysis, where distinct Laue oscillations were observed. The strong intensity oscillations revealed crystalline and well-ordered nickel oxide and magnetite films with homogeneous thicknesses on both substrates.

The vertical layer distances of all NiO films showed no dependence on the thickness in the investigated range. However, NiO and Fe_3_O_4_ films grown on MgO exhibited a vertical compressive strain while NiO and Fe_3_O_4_ films on SrTiO_3_ showed vertical tensile strain due to lattice matching at the interface. Based on elastic theory for continuum, the vertical lattice constant *c* for homogenous tetragonally (in-plane) distorted films is related to the lateral lattice constant *a* via [23]

(1) $$\begin{array}{c} \frac{\Delta c}{c}=\frac{2\nu }{\nu -1}\frac{\Delta a}{a}. \end{array}$$

**Figure 6:**
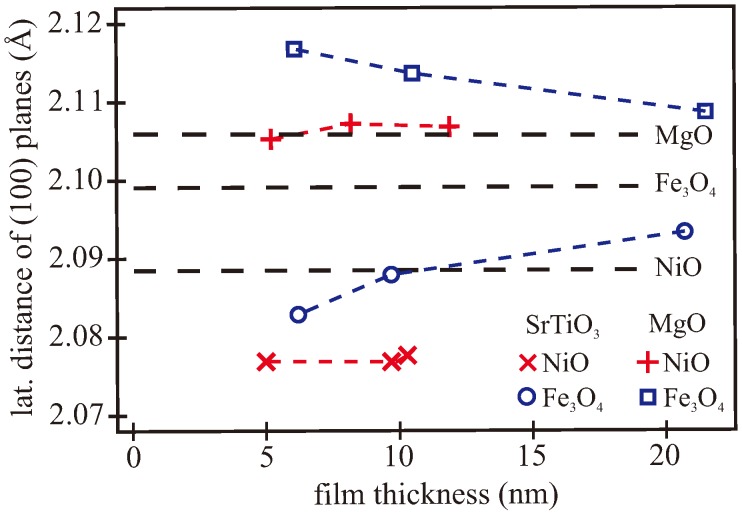
Lateral distance of (100) planes of all prepared magnetite and nickel oxide films calculated using Equation (1) and the vertical layer distances obtained from XRD analysis. The dashed lines denote the fully relaxed bulk values of MgO, Fe_3_O_4_, and NiO.

For the calculation of the vertical layer distance for a completely strained film, Δa from pseudomorphic growth was used. Assuming a Poisson number of ν = 0.21 for NiO [20], the vertical layer distance of pseudomorphic nickel oxide on MgO was calculated to be 2.079 Å. Hence, the NiO films grown on MgO were fully strained as clarified by Figure 6 where lateral distances of (100) planes are presented.

Above a critical thickness $d_{c}$, this strain should reduce rapidly due to the stable formation of dislocations. Following the model of Matthews and Blakeslee [66], the critical thickness $d_{c}$, at which the generation of misfit dislocation will begin, can be calculated by the formula

(2) $$\begin{array}{c} \frac{d_{c}}{b}=\frac{\left(1-\nu \;\cos^{2}\alpha \right)\;\left(\ln\left(\frac{d_{c}}{b}\right)+1\right)}{2\pi f(1+\nu )\cos(\lambda )}. \end{array}$$

Here, *b* is the magnitude of the Burgers vector, *f* is the lattice mismatch, ν is the Poisson ratio, α = 90° is the angle between the Burgers vector and the dislocation line, and λ = 45° is the angle between the Burgers vector and the direction both normal to the dislocation line and within the plane of the interface. For NiO films on MgO(001), the critical thickness was determined to 39 nm. Since the studied films were below the critical thickness, the absence of strain relaxation is in good agreement with this model. Similar results were also observed by Schemme et al. [33] for NiO films of different thicknesses up to 34 nm grown on MgO(001). The experimental data of James et al. [20] showed a strain relaxation above ∼40 nm, which is consistent with our observations and confirms Equation (2).

Despite the large misfit of −6.9% between NiO and SrTiO_3_, the XRD curves of all studied films also featured distinct Laue oscillations, pointing to a good crystalline ordering. Assuming a complete lattice matching at the interface, we calculated a vertical lattice distance of 2.161 Å for fully strained NiO films on SrTiO_3_ (Equation (1)), while we observed a film thickness independent value of 2.095 Å. The resulting lateral distances of the (100) planes calculated by Equation (1) of all investigated nickel oxide and magnetite films are presented in Figure 6. Thus, for the NiO films grown on SrTiO_3_, the remaining lateral strain only amounted to −0.6% (cf. Figure 6). For the critical thickness, Equation (2) revealed a value of 3.5 nm. All prepared NiO films were well above the critical thickness. Thus, the observed strong strain relaxation seems to be reasonable although they were not completely relaxed. We assume that the residual strain cannot be removed from the film due to kinetic barriers preventing the film from relaxing completely. Similar strain behavior was reported by Zhang et al. for NiO films of 2 nm thickness grown by pulsed laser deposition on SrTiO_3_ substrates. In contrast to our findings, a complete relaxation for NiO films of thicknesses above 10 nm was observed, probably driven by higher deposition temperature [40].

In the case of Fe_3_O_4_ on NiO/MgO, we calculated a vertical layer distance for a fully strained film of 2.092 Å and a critical thickness of 105 nm (ν = 0.356 [67], *f* = 0.3%), applying Equations (1) and (2), respectively. Here, the misfit *f* coincided with the misfit of magnetite on MgO since the growth of NiO on MgO was pseudomorphic adapting its lateral lattice constant (cf. Figure 6). All our investigated magnetite films on NiO/MgO were strongly strained, having a lower vertical layer distance than received by Equation (1). Further, the calculated lateral layer distance of all prepared Fe_3_O_4_ films was larger than that of the NiO films pseudomorphically grown on MgO (cf. Figure 6). Consequently, the magnetite films were exposed to much higher tensile lateral strain as expected from classical growth theory. This effect may be attributed to the unpreventable formation of APBs, which is not considered in the simple theories of epitaxial growth and relaxation via misfit dislocations. Thus, we assume that APBs expose additional tensile strain to the magnetite film. This result is in contrast to the compressive strain due to APBs as reported for magnetite films (thickness range 85–600 nm) directly grown on MgO(001) by magnetron sputtering [68]. As shown in Figure 4c, the measured vertical layer distance approached the bulk value with increasing Fe_3_O_4_ thickness. However, the bulk value was not reached even for the thickest magnetite film on NiO/MgO studied here. On one hand, this effect is very surprising since the predicted critical thickness of 105 nm is beyond the thicknesses under consideration here. On the other hand, this behavior of partial relaxation below the calculated critical thickness also coincides with the results reported by Schemme et al. [33]. We also attribute this effect to the unpreventable formation of APBs, which is not considered in the simple theories for the nucleation of misfit dislocations. Thus, APBs seem to lower the kinetic barrier for the formation of dislocations.

Regardless of the low remaining compressive strain between the Fe_3_O_4_ and NiO/SrTiO_3_, these magnetite films were less structurally ordered than the magnetite films grown on NiO/MgO. While the crystalline quality of the NiO films on SrTiO_3_ was constantly high independent of the film thickness, the strength of Laue oscillations of the Fe_3_O_4_ films grown on top of NiO/SrTiO_3_ decreased with increasing magnetite thickness. This result is supported by the high surface roughness of the magnetite films obtained from the XRR measurements as well as by the broadened diffraction spots seen in the LEED pattern.

Assuming the pseudomorphic growth of magnetite on the strained NiO film with remaining lattice mismatch of −1%, the vertical layer distance of a fully strained magnetite film was calculated to be 2.123 Å using Equation (1). The measured value of 2.117 Å for the 5 nm-thick magnetite film was already lower than the expected value for pseudomorphic growth (cf. Figure 4c). Thus, this magnetite film was already partially relaxed and showed vertical and lateral strain of 0.9% and 0.8%, respectively (cf. Figure 6). With increasing thickness of the magnetite film, the vertical and lateral layer distances strongly relaxed further to 2.104 Å and 2.093 Å, respectively, for the 20.7 nm-thick film. Again, this effect contradicts classical relaxation theory via dislocation formation from which the critical film thickness of 27 nm was obtained using Equation (2). Consequently, the formation of grain boundaries and structural defects (e.g., APBs) during the initial stage of film growth may support the formation of misfit dislocations, and thus a faster relaxation process. In addition, as stated above, the lateral tensile strain due to APBs may cause a larger lateral layer distance compared to pseudomorphic growth on the strained NiO film.

VSM measurements of the two thickest magnetite films on NiO/MgO and NiO/SrTiO_3_ revealed ferro(i)magnetic behavior for both samples. However, the Fe_3_O_4_ film grown on NiO/SrTiO_3_ showed enhanced coercive field compared to the film on NiO/MgO, possibly caused by a higher density of grain boundaries, and thus the formation of more pinning centers as confirmed by the LEED analysis. This behavior coincides with the weaker structural ordering and higher surface roughness of the magnetite films on NiO/SrTiO_3_, also seen in the XRD and XRR measurements. An increased coercive field for magnetite films grown on SrTiO_3_ caused by a higher surface roughness or strain has also been reported in Refs. [69,70].

The obtained saturation magnetization values of Fe_3_O_4_ grown on NiO/MgO and NiO/SrTiO_3_ coincided within the error tolerances with the values determined by XMCD [42]. Additionally, the value of Fe_3_O_4_ film on NiO/MgO was also rather close to the ideal theoretical value as well as to the experimental bulk moment of magnetite of 4.07 $\mu_{B}$/f.u. [5,63,71], whereas Fe_3_O_4_ on NiO/SrTiO_3_ exhibited a lower value. A reduced magnetic moment has also been reported for Fe_3_O_4_/SrTiO_3_ systems, possibly caused by a large density of APBs induced by high lattice mismatch [34,72]. This result is supported by a weaker structural ordering as well as higher coercive fields and, thus, a higher density of grain boundaries observed for Fe_3_O_4_ on NiO/SrTiO_3_.

Further, both investigated samples showed a fourfold magnetic in-plane anisotropy with magnetic easy axes aligned along the $\langle 100\rangle$ directions. For thin magnetite films on MgO(001), the magnetic easy axes are mostly reported to point into $\langle 110\rangle$ directions [70,73,74] as expected from bulk properties of Fe_3_O_4_. However, a magnetic isotropic behavior [74,75] or magnetic easy axes aligned in $\langle 100\rangle$ directions [76] are also presented in the literature for Fe_3_O_4_/MgO(001). Moreover, magnetite films grown on an iron buffer layer deposited on MgO(001) also exhibited a magnetic in-plane anisotropy with magnetic easy axes parallel to $\langle 100\rangle$ [77]. For Fe_3_O_4_ films on SrTiO_3_(001), different orientations of the magnetic easy axes were also reported. While Kale et al. observed a fourfold magnetic anisotropy with magnetic easy axes pointing into $\langle 110\rangle$ directions [75], magnetic easy axes aligned along the $\langle 100\rangle$ directions are presented in Refs. [35,76]. All these observations show that the magnetic properties of magnetite are highly affected by the interface between the film and substrate and can be influenced by the deposition conditions, lattice mismatch, or stoichiometric deviations. In addition, we assume that a tetragonal distortion of the films can influence the spin–orbit coupling, which may lead to modified magnetocrystalline anisotropy constants [78] and, thus, altered directions of magnetic easy and hard axes.

## 5. Conclutions

We present a comparative study on the structural and magnetic properties of Fe_3_O_4_/NiO bilayers grown on MgO(001) and Nb-doped SrTiO_3_(001). Stoichiometric magnetite and NiO films with homogenous thicknesses were found on both substrates in the investigated thickness range (5–20 nm). Detailed analysis of the XRD measurements revealed a high crystallinity of the NiO films independent of the underlying substrate or film thickness. However, magnetite films grown on NiO/SrTiO_3_ showed a weaker structural ordering and higher surface roughness compared to the films grown on NiO/MgO, induced by a large lattice mismatch and the resulting relaxation process. Further, the bilayers exhibited a vertical compressive strain on MgO but a tensile strain in the vertical direction on SrTiO_3_ as a result of lateral compression. The weaker crystalline structure of Fe_3_O_4_ on NiO/SrTiO_3_ affected the magnetic properties, leading to an enhanced coercive field and a reduced magnetic moment compared to magnetite on NiO/MgO. Nevertheless, these Fe_3_O_4_/NiO bilayers on MgO and SrTiO_3_ substrates are expected to show large thermoelectric effects based on the thermal generation of spin currents (spin Seebeck effect) [11,12,13], supported by the antiferromagnetic NiO layer [21,22].

Additionally, both systems showed a fourfold magnetic in-plane anisotropy with magnetic easy axes pointing in $\langle 100\rangle$ directions which were 45° rotated to the well-known magnetic easy axes directions of thin magnetite films on MgO(001) as expected from bulk properties. One potential reason may be a modified spin–orbit coupling as a result of the tetragonal distortion of the films leading to altered magnetocrystalline anisotropy. A detailed understanding of these bilayers is of the utmost importance since they are excellent candidates for potential spintronic and spin caloritronic applications. Therefore, this behavior deserves further study to shed more light on this interesting change of the magnetic anisotropy of Fe_3_O_4_ thin films grown on NiO/MgO(001) and NiO/SrTiO_3_(001).
